# Venetoclax and Azacitidine in the Treatment of *NPM1*-Mutated Donor Cell–Derived Leukemia in a Patient With Fanconi Anemia: Case Report and Literature Review

**DOI:** 10.1200/PO.22.00693

**Published:** 2023-06-14

**Authors:** Julie Ma, Kenji Morimoto, Michael A. Pulsipher, Chintan Parekh

**Affiliations:** ^1^ Cancer and Blood Disease Institute, Children's Hospital Los Angeles, Los Angeles, CA; ^2^ Department of Pediatrics, Kaiser Permanente Fontana Medical Center, Fontana, CA; ^3^ Division of Pediatric Hematology and Oncology, Intermountain Primary Children's Hospital, Huntsman Cancer Institute, University of Utah, Salt Lake City, UT; ^4^ Department of Pediatrics, Keck School of Medicine, University of Southern California, Los Angeles, CA

## Introduction

Fanconi anemia (FA), a genetic DNA damage repair (DDR) pathway deficiency, is the most common inherited bone marrow (BM) failure syndrome.^1^ Features of FA include chromosomal instability, congenital abnormalities, extreme sensitivity to DNA-damaging agents, and a predisposition for malignancy, particularly myelodysplastic syndrome (MDS), acute myeloid leukemia (AML), and solid tumors.^2-4^

Allogeneic hematopoietic stem-cell transplantation (allo-HSCT) constitutes curative therapy for BM failure, MDS, and AML in FA,^5^ but importantly, the nonhematopoietic cells remain unable to repair DNA damage post-transplant.^6^ Typical intensive AML chemotherapy regimens carry an unacceptably high risk of toxicity in patients with FA, including prolonged myelosuppression and mucositis.^7^ FA-associated MDS/AML is treated with reduced intensity conditioning (RIC) HSCT preceded by no or limited chemotherapy because of the extreme sensitivity of FA cells to alkylating agents and radiation,^5,8^ higher rates of graft-versus-host disease,^9,10^ and predisposition to solid malignancies.^10-12^ Of note, given the presence of the FA DDR deficiency in the MDS/AML cells, FA-associated MDS/AML is sensitive to low-intensity chemotherapy.^13^

Donor cell–derived leukemia (DCL) is a complication of allo-HSCT where leukemia arises from donor cells after engraftment and has a poor prognosis.^14-16^ The etiology underlying DCL is likely multifactorial; proposed factors include germline mutations and rapid proliferation of donor cells, post-transplant immunocompromised state, and treatment damage to the BM microenvironment.^14,16-18^ A multiple-hit hypothesis where hematopoietic stem cells with a pre-existing defect are influenced by recipient- and transplant-related factors to transform into malignancy is supported by the lack of leukemogenesis in the donor in most cases.^15-18^

Here, we report a case of a child who developed donor-derived *NPM1*-mutated MDS/AML after HSCT for BM failure because of FA. Management of DCL in a patient with FA posed a unique challenge because of the need for a more effective approach than RIC without causing excessive toxicity. Since donor-derived myeloblasts would not be as sensitive to chemotherapy as the DDR-deficient blasts in FA-related MDS/AML, RIC alone would likely not induce a remission. However, intensive chemotherapy would be highly toxic in a patient in whom all the nonhematopoietic cells carried the FA defect. Therefore, she was treated with venetoclax/azacitidine, a regimen with minimal nonhematopoietic toxicity and low DNA damage potential known to induce high remission rates in *NPM1*-mutated AML, before a second RIC HSCT. The patient's guardian consented to the submission of the case report to the journal.

## Case Report

A 5-year-old girl was diagnosed with FA after presenting with thrombocytopenia. BM showed hypocellularity (20%-40%) without dysplasia and normal cytogenetics. BM failure testing was negative for paroxysmal nocturnal hemoglobinuria (flow cytometry) with normal telomere lengths and serum adenosine deaminase levels. FA was diagnosed on the basis of abnormal chromosome breakage with diepoxybutane and mitomycin C and a compound heterozygous *FANCA* mutation (Table 1).^2^

**TABLE 1.**
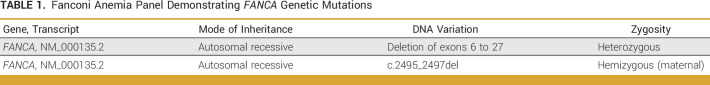
Fanconi Anemia Panel Demonstrating *FANCA* Genetic Mutations

She underwent a 4/6 matched cord blood HSCT preceded by RIC with low-dose cyclophosphamide (10 mg/kg daily for 4 days), fludarabine (35 mg/m^2^ daily for 4 days), total body irradiation (300 cGy), and antithymocyte globulin (ATG). She experienced engraftment after 13 days with resolution of BM failure. Forty-five months after HSCT, she developed thrombocytopenia (9k/uL). BM showed myelodysplasia with 10% myeloblasts and 100% donor chimerism, consistent with DCL. *NPM1*, *GATA1*, and *WT1* mutations were detected, indicative of donor-derived MDS/AML (Table 2).^19,20^

**TABLE 2.**
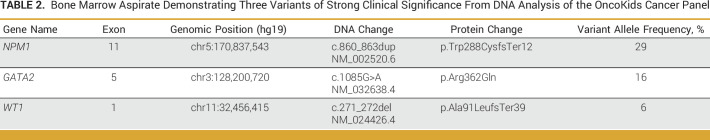
Bone Marrow Aspirate Demonstrating Three Variants of Strong Clinical Significance From DNA Analysis of the OncoKids Cancer Panel

Given the high risk of toxicity with DNA-damaging agents in FA and the high sensitivity of myeloblasts with the FA DDR defect, FA-related MDS/AML is typically treated with RIC HSCT without intensive chemotherapy before conditioning.^21-25^ However, in view of the blasts being of donor origin (ie, no chemotherapy-sensitizing FA DDR defect) and the planned use of RIC for a second HSCT, she was considered to be at high risk for post-transplant relapse without remission-inducing antileukemia therapy before conditioning. A pretransplant minimal residual disease (MRD)–negative remission is one of the strongest predictors of favorable outcomes for HSCT in AML.^26^ In AML, *NPM1* mutations are strongly predictive of a high MRD-negative response rate (88%) with venetoclax/azacitidine therapy.^27-29^ Furthermore, the low DNA-damaging potential of venetoclax/azacitidine was favored given the exquisite sensitivity of the nonhematopoietic FA cells to DNA-damaging agents. Thus, the toxicity risks related to the FA DDR defect, chemotherapy mechanisms of action, and specific molecular profile of the MDS/AML drove the choice of venetoclax/azacitidine.

Azacitidine was administered (75 mg/m^2^ dose once daily) for 1 week, and venetoclax was administered for 21 days instead of the typical 28 days to limit myelosuppression.^30,31^ The US Food and Drug Administration–approved adult dose of venetoclax with the recommended package insert reduction for concomitant posaconazole prophylaxis was used (Table 3).^32-34^ She was admitted for observation because of grade 4 neutropenia. After one course of venetoclax/azacitidine, she developed a subdural hematoma of unknown etiology (platelet count >100k/uL). She underwent craniotomy and evacuation; HSCT was delayed by 1 month. BM showed complete remission with MRD negativity by flow cytometry and *NPM1* polymerase chain reaction (PCR). She subsequently contracted respiratory syncytial virus, necessitating a further 6-week delay of HSCT. Given the delay, an additional 14-day cycle of venetoclax/azacitidine was administered, a duration demonstrating similar efficacy and reduced myelosuppression relative to the conventional 28-day cycle in a study of elderly patients with AML.^35^ After the second cycle, the BM showed MRD negativity by flow cytometry; peripheral blood showed low level *NPM1* PCR positivity (ratio of 0.0056). She then underwent RIC with low-dose cyclophosphamide, fludarabine, busulfan, and ATG followed by an 8/10 cord blood HSCT. BM was MRD-negative (flow cytometry and PCR) 3 months post-HSCT. She remains in remission over 1.5 years after HSCT.

**TABLE 3.**
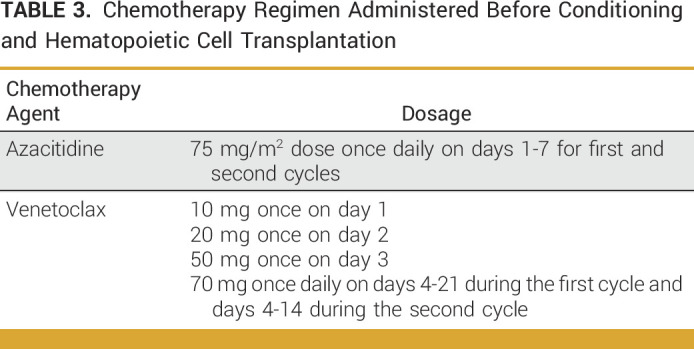
Chemotherapy Regimen Administered Before Conditioning and Hematopoietic Cell Transplantation

## Discussion

DCL is a rare complication of allo-HSCT and, to our knowledge, has not been reported in a host with FA.^36^ Because of its poor prognosis, our patient was thought to require more potent antileukemia therapy than RIC alone to maximize the probability of a pretransplant MRD-negative remission and thereby optimize the likelihood of a curative second RIC HSCT. Standard conditioning regimens yield exceedingly poor outcomes in FA.^8^ Thus, HSCT in FA is now performed with RIC regimens; these have resulted in improved outcomes and decreased toxicity.^7^ The high risk of toxicity precludes the use of intensive chemotherapy before HSCT conditioning in FA-related AML; chemotherapy before conditioning is limited to low-dose regimens.^13,37,38^

Hematologic malignancies highly express the antiapoptotic protein B-cell leukemia/lymphoma-2 (BCL2).^39^ Venetoclax, a selective BCL2 inhibitor, counteracts BCL2-mediated cell survival.^39^ Azacitidine, a DNA hypomethylating agent, synergizes with venetoclax to induce apoptosis through NOXA, a proapoptotic protein.^40^ The combination of venetoclax with a hypomethylating agent has demonstrated promising outcomes without significant toxicity in MDS/AML (67% composite response rate [CRR, complete remission with or without count recovery], 29% MRD negativity rate).^41,42^ Of note, the presence of an *NPM1* mutation is strongly predictive of response (91.5% CRR, odds ratio 5).^41^ Several laboratory studies have uncovered potential mechanisms by which mutant *NPM1* increases sensitivity to venetoclax. *NPM1*-mutated AML cells show 20-fold higher sensitivity to venetoclax than *NPM1* wild-type AML cells in vitro.^43^ *HOX* overexpression, a key leukemogenic mechanism in *NPM1*-mutated cells,^44^ is strongly associated with increased sensitivity to venetoclax in ex vivo drug sensitivity studies.^45^ Venetoclax in combination with azacitidine eliminates leukemic stem cells by inhibiting mitochondrial electron transport chain complex II activity.^46^ Two studies have shown that inhibition of mitochondrial function sensitizes AML cells to venetoclax.^47,48^ Studies in mice and cell lines have demonstrated that mutant *NPM1* impairs mitochondrial function, a likely mechanism for the exquisite sensitivity of *NPM1*-mutated cells to venetoclax.^48^ Venetoclax/azacitidine induced a morphologic response in six of eight children with MDS or AML; three were MRD-negative.^49^ To our knowledge, the use of venetoclax/azacitidine to achieve a deep remission in MDS/AML in FA or DCL has not been reported.

AML is distinguished from MDS on the basis of a BM blast cutoff of >20%. Several myeloid neoplasms are classified as AML on the basis of the presence of an AML-defining genomic aberration [eg, t(8; 21)], regardless of the blast percentage. In the current WHO classification, *NPM1* mutation is not adequate for the diagnosis of AML when the blast percentage is below 20%. However, several studies suggest rapid progression to AML and a need for intensive AML-type therapy in MDS with *NPM1* mutations, raising the question of whether *NPM1*-mutated MDS should be classified as AML.^50^ Since MDS with excess blasts and AML are a continuum and *NPM1*-mutated MDS tends to rapidly progress to AML, we considered our patient to have MDS/AML and used an AML-type approach to achieve a deep remission before HSCT.

In conclusion, we report a unique scenario of *NPM1*-mutated DCL in a patient with FA where MRD-negative remission with limited toxicity was achieved using venetoclax/azacitidine before HSCT, and the patient remains disease-free for over a year after HSCT. This case suggests that venetoclax/azacitidine can induce a deep remission in children with *NPM1*-mutated myeloid DCL who cannot tolerate intense chemotherapy and may represent a useful approach in the setting of other DDR defects or telomere diseases (eg, dyskeratosis congenita) that are characterized by poor chemotherapy tolerance; these patients often undergo HSCT for BM failure and are thereby at risk for DCL.^3,51^ Studies such as the ongoing multicenter trial (ClinicalTrials.gov identifier: NCT05292664) of venetoclax/azacitidine are needed to investigate the outcomes and optimal dosing for this regimen in FA.
